# Malaria Diagnosis Using a Lightweight Deep Convolutional Neural Network

**DOI:** 10.1155/2022/4176982

**Published:** 2022-04-15

**Authors:** Varun Magotra, Mukesh Kumar Rohil

**Affiliations:** ^1^Department of Computer Engineering, Sardar Patel Institute of Technology, Andheri West, Mumbai, 400053 Maharashtra, India; ^2^Department of Computer Science and Information Systems, Birla Institute of Technology and Science, Pilani, 333031 Rajasthan, India

## Abstract

The applications of AI in the healthcare sector are increasing day by day. The application of convolutional neural network (CNN) and mask-region-based CNN (Mask-RCCN) to the medical domain has really revolutionized medical image analysis. CNNs have been prominently used for identification, classification, and feature extraction tasks, and they have delivered a great performance at these tasks. In our study, we propose a lightweight CNN, which requires less time to train, for identifying malaria parasitic red blood cells and distinguishing them from healthy red blood cells. To compare the accuracy of our model, we used transfer learning on two models, namely, the VGG-19 and the Inception v3. We train our model in three different configurations depending on the proportion of data being fed to the model for training. For all three configurations, our proposed model is able to achieve an accuracy of around 96%, which is higher than both the other models that we trained for the same three configurations. It shows that our model is able to perform better along with low computational requirements. Therefore, it can be used more efficiently and can be easily deployed for detecting malaria cells.

## 1. Introduction

Malaria is caused by the plasmodium parasite, and this parasite is transmitted to a healthy person by the bite of a mosquito carrying the parasite. Millions of people get infected by malaria every year. For example, in the year 2019, there were 229 million cases of malaria all over the world, out of which 409,000 people died. A lot of research is being done by different countries and WHO to fully eradicate malaria from the world. Many countries have been able to achieve this, but there are a lot of countries where malaria is still a very serious health problem.

Various efforts are being made to diagnose malaria easily and efficiently [1, 2]. The traditional method involves procuring the blood sample of the patient and taking it to the laboratory where a skilled and experienced health professional is required to discriminate between the healthy and parasitic red blood cells (RBCs). This process of identification is a very tedious and time-consuming task. So, to reduce the cost of blood tests and lab technicians for testing the malaria parasites in the blood sample, it is advised to use a cost-effective digitized approach in one or other forms to detect the parasitic cell to diagnose malaria.

Deep learning methodologies are being explored for their suitability to be applied to the diagnosis of various diseases including malaria. Further in this paper, we explore some of these methodologies and explain how the current algorithms are being used to improve the accuracy at minimum possible hardware cost so as to ease the process of identifying diseases at a reasonably reduced cost.

Further, the content of the article is organized as follows: we discuss some related work in Section 2. We describe the dataset and the proposed model in Section 3.  Section 4 presents details on the training of the proposed model and the experimentation performed. In Section 5, we bring out the results and discuss the observations in detail. Finally, Section 6 furnishes the concluding remarks to the work.

## 2. Related Work

In recent years, due to the scalability of deep learning methods and robustness of new machine learning models, these have proven to be very useful in providing acceptable solutions in the medical domain. The introduction of convolutional neural networks [3] was a breakthrough for computer vision and image processing tasks. CNN proved to be extremely useful in solving computer vision tasks like image classification, feature extraction, and medical image analysis. CNN provides an efficient way to capture the information and learning features of images with the help of filters, and then, this information can be fed to the feedforward neural nets or machine learning algorithms to perform the specific tasks.

CNN models have millions of parameters to capture the relevant information and feature details, which can be used to accomplish various tasks. After CNN became the mainstream network for most of the tasks relating to image processing and computer vision, many state-of-the-art models like Resnet-50 [4], VGG-19 [5], and Inception v3 [6] are based on the CNN architecture. Although these (state-of-the-art) models have proven to be useful and efficient in solving various computer vision tasks, training these models on a particular set of data takes too much time and is computationally quite expensive. The concept of transfer learning has become so popular in the last five years that it has been used in almost every computer vision application. For the identification and classification tasks, transfer learning has demonstrated improved performance and accuracy and so has gained further popularity [7]. In recent times, transfer learning has been applied to the malaria dataset to identify the parasitic plasmodium cells and segregate them from the healthy cells [2], and it is able to achieve good results on the data. Sarkar et al. [2] have proposed a three-layered CNN and compared its performance with two models. This three-layered CNN [2] achieves accuracy up to 95.80%.

In 2017, Dong et al. in their study used three SOTA models that are based on CNN architecture on detecting the malaria-infected cells [8]. The authors evaluated LeNet, AlexNet, and GoogleNet on the datasets and were able to achieve accuracy up to 95% showing how these deep learning models are able to perform better than the traditional machine learning approaches like SVM which achieved an accuracy of 92% in their study.

In 2020, Shekar et al. in their study proposed a CNN network for identifying the parasitic infected cell. Shekar et al. compared their model performance with the pretrained VGG-19 model and VGG-19 model which is trained on the malaria dataset [9] on which they trained their own model. The basic CNN model was able to achieve an accuracy of 94%, whereas the VGG-19 model trained on the same dataset was able to achieve an accuracy of 96% [10].

In solving image classification problems, many of the methods discussed have been successful and some of these are useful for malaria parasitic cell detection [1, 2, 5, 6]. In the present work, we first discuss the advantages, disadvantages, and limitations of each of these methods for malaria parasitic cell detection and classification, and then, we propose a new method that outperforms the two widely used methods in terms of speed while maintaining the classification accuracy at par with these methods.

## 3. Dataset and the Proposed Model

### 3.1. Dataset Description

The dataset, used for this comparative study, comprises microscopic images of RBCs and is available at the website of the National Library of Medicine (NLM), USA [9]. This dataset contains two classes, one containing the parasitic RBC images and the other containing healthy RBC cell images. Each class contains 13,794 images. For our experimentation, we partition the dataset into three different proportions (i.e., 30 : 70, 50 : 50, and 70 : 30) in training samples and validation samples, and we run our experiments for each of these partitions. The images have different resolutions. Therefore, to feed the images with the same resolution, we have resampled each image to 134 × 134 resolution.

### 3.2. Image Preprocessing and Data Augmentation

**Figure 1:**
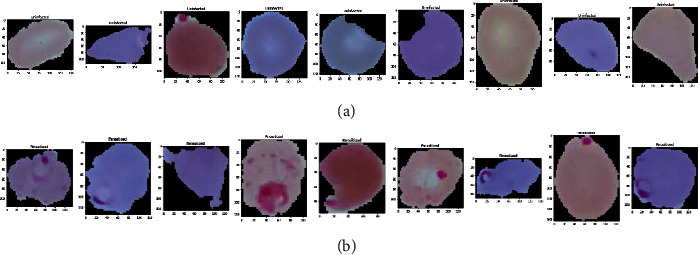
Some sample images of RBCs without augmentation: (a) uninfected and (b) parasitized.

**Figure 2:**
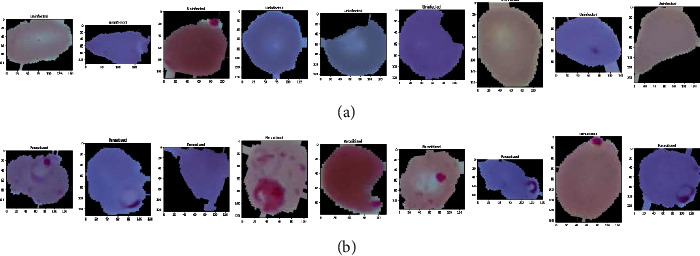
Some sample images of RBCs without augmentation: (a) uninfected and (b) parasitized.

In our experiment, we used Keras image data preprocessing to make data more augmented and diverse. We used parameters like rotational, shear and zoom ranges, width shift, height shift, and horizontal flip. To overcome the challenge posed by varied input image sizes, we used rescaling parameters to resize all the images to the dimensions of 134 × 134. To normalize the image gray-level values, we used a rescale parameter, and we employed a data-split generator to split the original data into testing and training of different sizes in various proportions.  Figure 1 depicts the RBCs without augmentation, and Figure 2 depicts the RBCs after augmentation.

### 3.3. Approach

#### 3.3.1. Custom CNN

**Figure 3:**
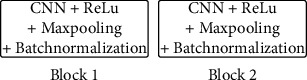
Fundamental CNN architecture configuration.

**Figure 4:**
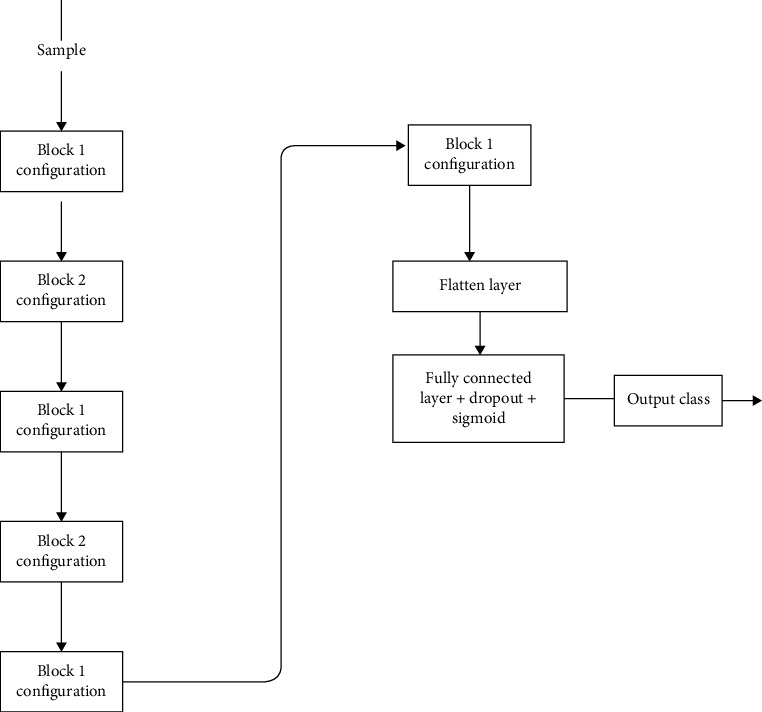
Custom CNN architecture overview.

In our study, we have created a customized CNN with six convolutional layers and the filter size ranging from 16 to 128 followed by the use of the “Relu” as an activation function and six max-pooling layers. We have used a dropout function thrice at different instances so that the overfitting of the model can be reduced during the training phase, thereby making the model more robust in real-life applications. To stabilize the learning, we applied batch normalization thrice. After flattening the output from the convolutional layer, two dense layers, with different neuron sizes and without any activation function, have been used. As the last layer, one dense layer has been added with only a single neuron to decide whether the given input is parasitic or healthy (i.e., uninfected), and a “sigmoid” function is used for activation. The total number of parameters in the custom CNN is 332,577, out of which trainable parameters (which are optimized and updated during training) are 332,161 and nontrainable parameters (which are not optimized and updated during training) are 416. The architecture is illustrated in Figures 3 and 4.

#### 3.3.2. Transfer Learning

For this purpose, we used two state-of-the-art models: one being “Inception v3” [6] and the other one “VGG-19” [5]. In this study, we use the weights as obtained as pretrained on the ImageNet dataset [11]. For both the networks, the top layers are adjusted to be untrainable, and a few layers are added to both of these networks as required. The basic framework of transfer learning is explained as follows:

For a given domain *D*, where a domain *D* is defined as follows:

(1) $$\begin{array}{c} D=\left\{\chi ,P\left(X\right)\right\}, \end{array}$$

where *χ* is the feature space and *P*(*X*) is the marginal distribution over *X* = {*x*_1_, ⋯, *x*_*n*_}, *x*_*i*_ ∈ *χ*.A task “*T*” is defined by two components as follows:

(2) $$\begin{array}{c} T=\left\{l,P\left(\frac{Y}{X}\right)\right\}=\left\{l,f\left(n\right)\right\}, \end{array}$$

where *Y* = {*y*_1_, ⋯, *y*_*n*_}, *y*_*i*_ ∈ *l*, *l* is a label space, and *f*(*n*) is a predictive function learned from feature vector/label pairs (*x*_*i*_, *y*_*i*_).

For each feature vector in the domain *D*, *f*(*n*) predicts its corresponding label as follows:

(3) $$\begin{array}{c} f\left(x_{i}\right)=y_{i}. \end{array}$$

Thus, transfer learning helps us accomplish various tasks pertaining to various domains without retraining the entire model from scratch and by retaining the knowledge learned from the previous tasks.

For the purpose of our research, the layer that we have added is just one, and the output of the untrainable layers is flattened by this layer. Further, two dense layers are used with neuron sizes 512 and 1024, respectively, with “Relu” as the activation function. The last layers of both the networks are the same as those of the custom CNN, with the same layer parameters and same activation functions. The reason for using the “Relu” as the activation function in all the models is that it provides nonsaturation of the gradient which gives an edge over the traditional activation functions like sigmoid and tanh.

The Relu function returns the 0 if the input from the layers is negative while returning the same value for any input that is positive. The formula for the same has been illustrated as follows:

(4) $$\begin{array}{c} f\left(x\right)=\max\left(0,x\right). \end{array}$$

The number of total parameters, the number of trainable parameters, and the number of nontrainable parameters of the VGG-19, Inception v3, and our proposed model are given in Table 1.

## 4. Training and Experimentation

The following steps have been carried out:

- Step 1: Once the model is initialized, the training is done with varying ratios of the amount of data for training and validation. The range for the size of training data varied from 30% to 70% of the complete data for three different iterations
- Step 2: Various callbacks have been used to make the experiment and the model more robust so that experiment does not proceed with further training without getting any increase in the performance. For this purpose, three callback methods “ModelCheckPoint,” “ReducLROnPlateau,” and “EarlyStopping” have been used as follows:

- Step 2.1: To identify the circumstances when checkpoint is created and to checkpoint the weights learnt, “ModelCheckPoint” class is used. As a result, our model achieves better weights and accuracy after the completion of the training phase
- Step 2.2: If the model has stopped improving its performance, “ReducLROnPlateau” is used to reduce the learning rate because a learning model often improves if it is programmed to reduce (by a factor of two to ten) the learning rate once learning does not improve the accuracy further in the subsequent and recent epochs. This callback is used to monitor the efficiency of the model, and if no improvement is observed in the efficiency for the number of epochs, the learning rate is reduced
- Step 2.3: Since a model can lead to overfitting if, without an increase in the performance, the model continues to learn for a long time, these types of situations are avoided by using “EarlyStopping”

(iii) Step 3: The optimizer “Adam” is used with the default learning rate (i.e., 0.001) for the custom CNN (i.e., the proposed model). The reason behind choosing “Adam” as the optimizing function is that Adam's default parameter configuration works well with most of the data, and also, the Adam takes into consideration the best properties of the AdaGrad and RMSProp algorithms to provide an efficient optimization algorithm.

The optimizer “RMSprop” has been used (with default learning rate, i.e., 0.0001) for the Inception v3 model and VGG-19. The “binary cross entropy” is used as a loss function for the three models and 32 images from a batch.

## 5. Results

**Figure 5:**
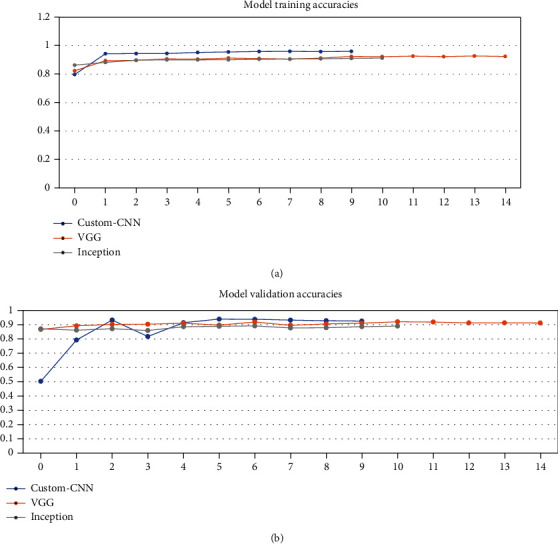
Classification accuracies obtained by the proposed model when dataset is partitioned in the ratio 30 : 70 into (a) training samples and (b) validation samples.

**Figure 6:**
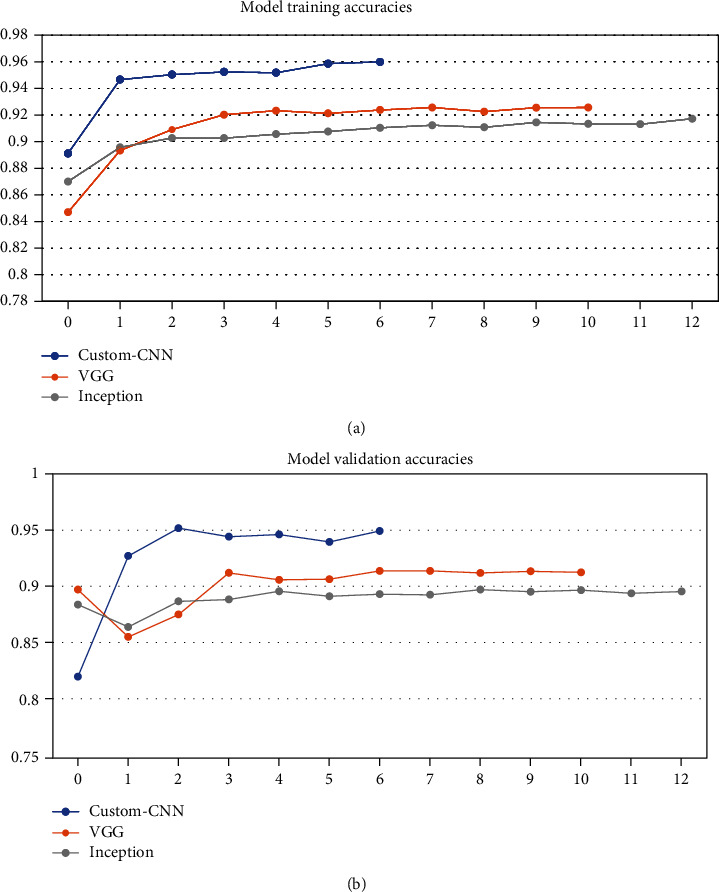
Classification accuracies obtained by the proposed model when dataset is partitioned in the ratio 50 : 50 into (a) training samples and (b) validation samples.

**Figure 7:**
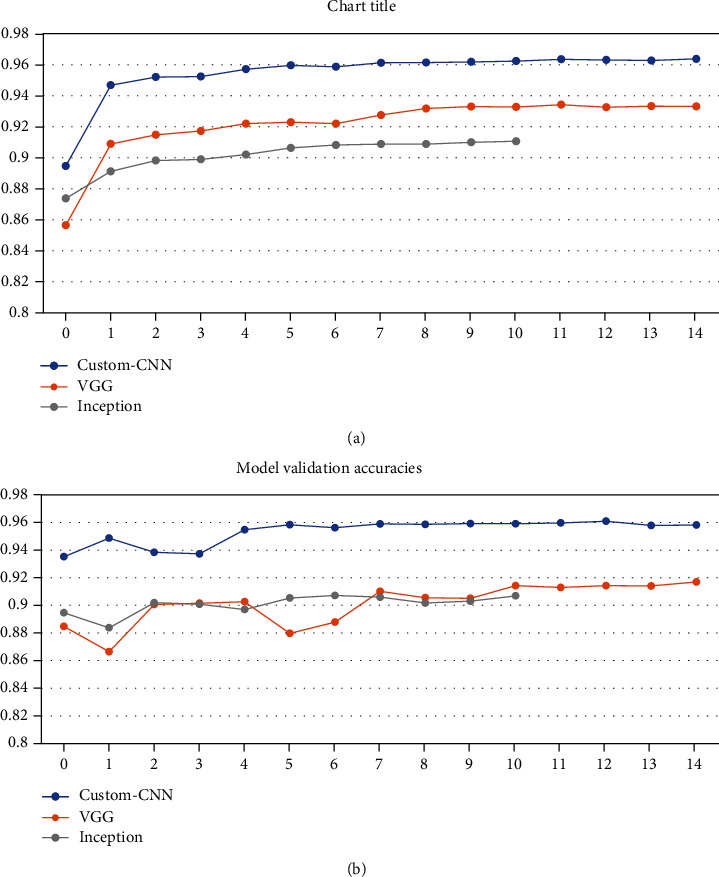
Classification accuracies obtained by the proposed model when dataset is partitioned in the ratio 70 : 30 into (a) training samples and (b) validation samples.

After training the models on data partitioned in varying ratios, each model got different training accuracy and validation accuracy running for the different number of epochs because of the “EarlyStopping” checkpoint as given in Table 2.  Figure 5 shows the classification accuracies obtained by the proposed model when dataset is partitioned in the ratio 30 : 70 into training samples and validation samples, respectively.  Figure 6 shows the classification accuracies obtained by the proposed model when dataset is partitioned in the ratio 50 : 50 into training samples and validation samples, respectively.  Figure 7 shows the classification accuracies obtained by the proposed model when dataset is partitioned in the ratio 70 : 30 into training samples and validation samples, respectively.

Out of all the models, our proposed CNN gave the best training as well as validation accuracy on all variations of data. The low difference in the training accuracy and the validation shows that our model has not been overfitted. The model is trained for 15 epochs for each of the data variations with the appropriate callbacks applied as explained in the previous section. The VGG-19 model gave the maximum training accuracy of 93.34 and validation accuracy of 91.64 when the data variation is 70 : 30. The difference between the training and validation accuracy is less which suggests that this model has not overfit on the data. The VGG-19 is trained for 15 epochs as our custom CNN, for all the data variation with all the callbacks applied. The Inception v3 performed the worst on the given data, giving the worst performance on all variation of data. The model is trained for epochs ranging from 5 to 14 on different variations of data with callback applied. It is trained in fewer epochs as the callbacks are applied, and the model accuracy does not increase on consecutive epochs, so it stopped the model from training further to prevent it from overfitting.

For further evaluation of our model, we evaluated the model on three parameters, i.e., precision, recall, and *F*1 score as shown in Table 3.

From Table 3, we can easily see how our proposed custom CNN model is having better precision, recall, and *F*1-score for every variation of data over VGG-19 and Inception v3. Therefore, in our study, our custom CNN which has lesser parameters than these CNN-based models is able to outperform them in detecting the parasitic malaria cells.

## 6. Conclusion

Malaria has been plaguing the world for many decades now. Hence, in our study, we proposed a custom CNN architecture to detect the malaria cells, and our proposed model is able to perform better than VGG-19 and Inception v3. Our proposed model not only has better accuracy than the VGG-19 and Inception v3 but also has lesser parameters than these models owing to which our model requires less computational power and a lesser number of iterations to train as compared to these models. As is well known that the models for medical purposes should be as compact as possible since they need to be deployed while hosting the large models is a big issue, therefore our approach has taken this problem into account and we are able to make the model as compact as possible without sacrificing accuracy. This study further consolidates the fact that the application of AI to healthcare industries is limitless and shall make a lot of work in the medical domain more economical since these kinds of automated models will be able to detect various diseases much more efficiently and economically.

## Figures and Tables

**Table 1:** Number of parameters (trainable and nontrainable) of all the three models.

Model	Total parameters	Trainable parameters	Nontrainable parameters
VGG-19	21,600,321	1,575,937	20,024,384
Inception v3	24,951,585	3,148,801	21,802,784
Our proposed model	381,729	381,313	416

**Table 2:** Accuracy comparison of all the three models on three different data configurations.

Data variation (%)	Accuracy (%)			Reference figure
	Proposed model (custom CNN)	VGG-19	Inception v3	
Training: 30	95.93 (training)	91.98 (training)	91.29 (training)	Figure 5
Validation: 70	95.20 (testing)	89.99 (testing)	88.83 (testing)	

Training: 50	95.73 (training)	93.05 (training)	91.66 (training)	Figure 6
Validation: 50	94.8 (testing)	91.99 (testing)	89.83 (testing)	

Training: 70	96.36 (training)	93.34 (training)	91.06 (training)	Figure 7
Validation: 30	96.23 (testing)	91.64 (testing)	91.03 (testing)	

**Table 3:** Precision, recall, and *F*1-score comparison of all the three models.

Data variation (%)	Model	Class	Precision	Recall	*F*1-score
Training: 30 Validation: 70	Proposed model (custom CNN)	Parasitic	0.96	0.95	0.95
		Uninfected	0.95	0.96	0.95
	VGG-19	Parasitic	0.87	0.93	0.90
		Uninfected	0.92	0.87	0.89
	Inception v3	Parasitic	0.86	0.91	0.89
		Uninfected	0.91	0.85	0.88

Training: 50 Validation: 50	Proposed model (custom CNN)	Parasitic	0.93	0.95	0.94
		Uninfected	0.95	0.93	0.94
	VGG-19	Parasitic	0.85	0.96	0.90
		Uninfected	0.95	0.83	0.88
	Inception v3	Parasitic	0.81	0.94	0.87
		Uninfected	0.93	0.78	0.85

Training: 70 Validation: 30	Proposed model (custom CNN)	Parasitic	0.92	0.97	0.94
		Uninfected	0.97	0.92	0.95
	VGG-19	Parasitic	0.87	0.94	0.90
		Uninfected	0.93	0.86	0.90
	Inception v3	Parasitic	0.80	0.96	0.87
		Uninfected	0.95	0.76	0.84

## Data Availability

Data are available at Malaria Datasets. [Online] Available: https://ceb.nlm.nih.gov/repositories/malaria-datasets/.
